# Dacomitinib as a First‐Line Therapy for Advanced EGFR‐Mutated Non‐Small Cell Lung Cancer Without Brain Metastases: A Multicenter Retrospective Observational Study

**DOI:** 10.1002/cam4.71659

**Published:** 2026-02-27

**Authors:** Ping‐Chih Hsu, How‐Wen Ko, Li‐Chung Chiu, Shih‐Hao Huang, Chung‐Shu Lee, Yu‐Ching Lin, Scott Chih‐Hsi Kuo, Jia‐Shiuan Ju, Chin‐Chou Wang, Cheng‐Ta Yang

**Affiliations:** ^1^ Division of Thoracic Medicine, Department of Internal Medicine Chang Gung Memorial Hospital at Linkou Taoyuan City Taiwan; ^2^ Department of Medicine, College of Medicine Chang Gung University Taoyuan City Taiwan; ^3^ Department of Thoracic Medicine New Taipei Municipal TuCheng Hospital New Taipei City Taiwan; ^4^ Division of Thoracic Oncology, Department of Respiratory and Critical Care Medicine Chang Gung Memorial Hospital, Chiayi Branch Chiayi County Taiwan; ^5^ Department of Respiratory Care Chang Gung University of Science and Technology, Chiayi Campus Chiayi County Taiwan; ^6^ Division of Pulmonary & Critical Care Medicine Kaohsiung Chang Gung Memorial Hospital Kaohsiung City Taiwan; ^7^ Department of Internal Medicine Taoyuan Chang Gung Memorial Hospital Taoyuan City Taiwan; ^8^ Department of Respiratory Therapy, College of Medicine Chang Gung University Taoyuan City Taiwan

**Keywords:** common EGFR mutation, dacomitinib, epidermal growth factor receptor mutation, non‐small cell lung cancer (NSCLC), tyrosine kinase inhibitor

## Abstract

**Background:**

Real‐world evidence regarding the use of dacomitinib as a first‐line therapy for advanced non‐small cell lung cancer (NSCLC) with epidermal growth factor receptor (EGFR) mutations remains limited. This multicenter, retrospective cohort study aimed to evaluate the clinical outcomes of dacomitinib as a first‐line treatment in patients with untreated advanced EGFR‐mutant NSCLC without brain metastases.

**Patients and Methods:**

This retrospective analysis included 161 patients with stage IIIB/IV EGFR‐mutant NSCLC without brain metastasis at baseline who received first‐line dacomitinib between October 2020 and August 2023 at four Taiwanese cancer centers. The primary outcomes included the objective response rate (ORR), progression‐free survival (PFS), overall survival (OS), predictive risk factors for PFS, and adverse events (AEs).

**Results:**

The ORR was 64.0%, and the disease control rate (DCR) reached 91.3%. The median PFS was 20.93 months (95% CI: 17.55–24.32), and the median OS was 41.27 months (95% CI: 31.71–50.82). Multivariate analysis revealed that an Eastern Cooperative Oncology Group (ECOG) performance status (PS) ≥ 2, bone metastasis, and liver metastasis were independent predictors of shorter PFS. Among patients who experienced disease progression and underwent rebiopsy, the secondary T790M mutation rate was 50.6%. Most treatment‐related AEs were grade 1–2 and manageable.

**Conclusions:**

Dacomitinib demonstrated favorable efficacy and tolerability as a first‐line therapy in advanced NSCLC patients with common EGFR mutations (exon 19 deletion or L858R). A baseline ECOG PS ≥ 2 and the presence of bone or liver metastases were significantly associated with worse PFS, suggesting a need for additional therapeutic strategies in these subgroups.

## 1 Introduction

Epidermal growth factor receptor (EGFR) mutations are the most prevalent oncogenic driver mutations found in non‐small cell lung cancer (NSCLC) patients, especially adenocarcinoma patients from East Asia, with prevalence rates ranging from 45% to 55% [1, 2]. EGFR mutations were first identified in 2004 and were reported to be correlated with the treatment response to the first‐generation EGFR‐tyrosine kinase inhibitor (TKI) gefitinib [3, 4]. Most EGFR mutations (~90%) are caused by a substitution of leucine (L) with arginine at position 858 in the EGFR protein (L858R) and the deletion of a specific sequence within exon 19 (exon 19 deletion) [1, 2, 3, 4]. In a previous pivotal clinical trial (IPASS study), gefitinib was shown to have significantly better clinical treatment efficacy (measured by the objective response rate (ORR) and progression‐free survival (PFS)) than conventional chemotherapy among advanced EGFR‐mutated NSCLC patients [5]. Therefore, EGFR mutations have been defined as specific biomarkers for the use of EGFR‐TKIs in the treatment of NSCLC patients [1, 2, 3, 4, 5]. In the past two decades, EGFR‐TKIs have been developed across three generations and have been demonstrated to be effective treatments for NSCLC patients harboring EGFR mutations in numerous prospective clinical trials [5, 6, 7, 8, 9, 10, 11]. Despite the remarkable clinical efficacy of EGFR‐TKIs, most patients eventually experience disease progression due to acquired resistance, heterogeneous tumor biology, or suboptimal treatment tolerability in real‐world practice. Cancer cell signaling redundancy and pathway crosstalk have been recognized as important contributors to therapeutic resistance, highlighting the complexity of long‐term disease control in advanced NSCLC. Consequently, there is growing interest in exploring treatment strategies that optimize existing targeted therapies and improve clinical outcomes beyond those observed in controlled clinical trials [12, 13].

Dacomitinib is classified as a second‐generation EGFR‐TKI and exhibits irreversible covalent binding to the tyrosine kinase domains of the ErbB1 (EGFR), ErbB2, and ErbB4 receptors, leading to blockade across the pan‐ErbB receptor family [9, 14, 15]. In a previous prospective clinical trial (ARCHER 1050), dacomitinib was shown to yield an ORR of 75% [9, 15]. In the same clinical trial, dacomitinib was also shown to lead to significantly longer PFS (14.7 vs. 9.2 months) and overall survival (OS) (34.1 vs. 27.0 months) than gefitinib when administered to untreated advanced‐stage NSCLC patients with EGFR mutations. According to the results of the ARCHER 1050 trial, dacomitinib was approved for first‐line therapy of metastatic NSCLC patients with EGFR exon 19 deletion or L858R mutation in September 2018 [14, 15, 16].

Among the first‐ and second‐generation EGFR‐TKIs (gefitinib, erlotinib, afatinib, and dacomitinib), dacomitinib is the most recently approved EGFR‐TKI for first‐line therapy for advanced NSCLC patients with EGFR mutations [16]. Other EGFR‐TKIs (gefitinib, erlotinib, and afatinib) are widely used as standard therapies in real‐world clinical practice, and their clinical outcomes have been reported in numerous previous studies [17, 18, 19]. The use of dacomitinib as a first‐line treatment for advanced EGFR‐mutated NSCLC patients in real‐world clinical practice has rarely been reported. Therefore, this multicenter retrospective study aimed to examine the clinical outcomes of advanced EGFR‐mutated NSCLC patients who received dacomitinib as first‐line therapy.

## 2 Methods

### 2.1 Patients

The patients included in this retrospective analysis were screened and retrieved from the registered cancer center databases of Linkou, Kaohsiung, and Chiayi Chang‐Gung Memorial Hospitals (CGMHs) and New Taipei Municipal TuCheng Hospital.

**FIGURE 1:**
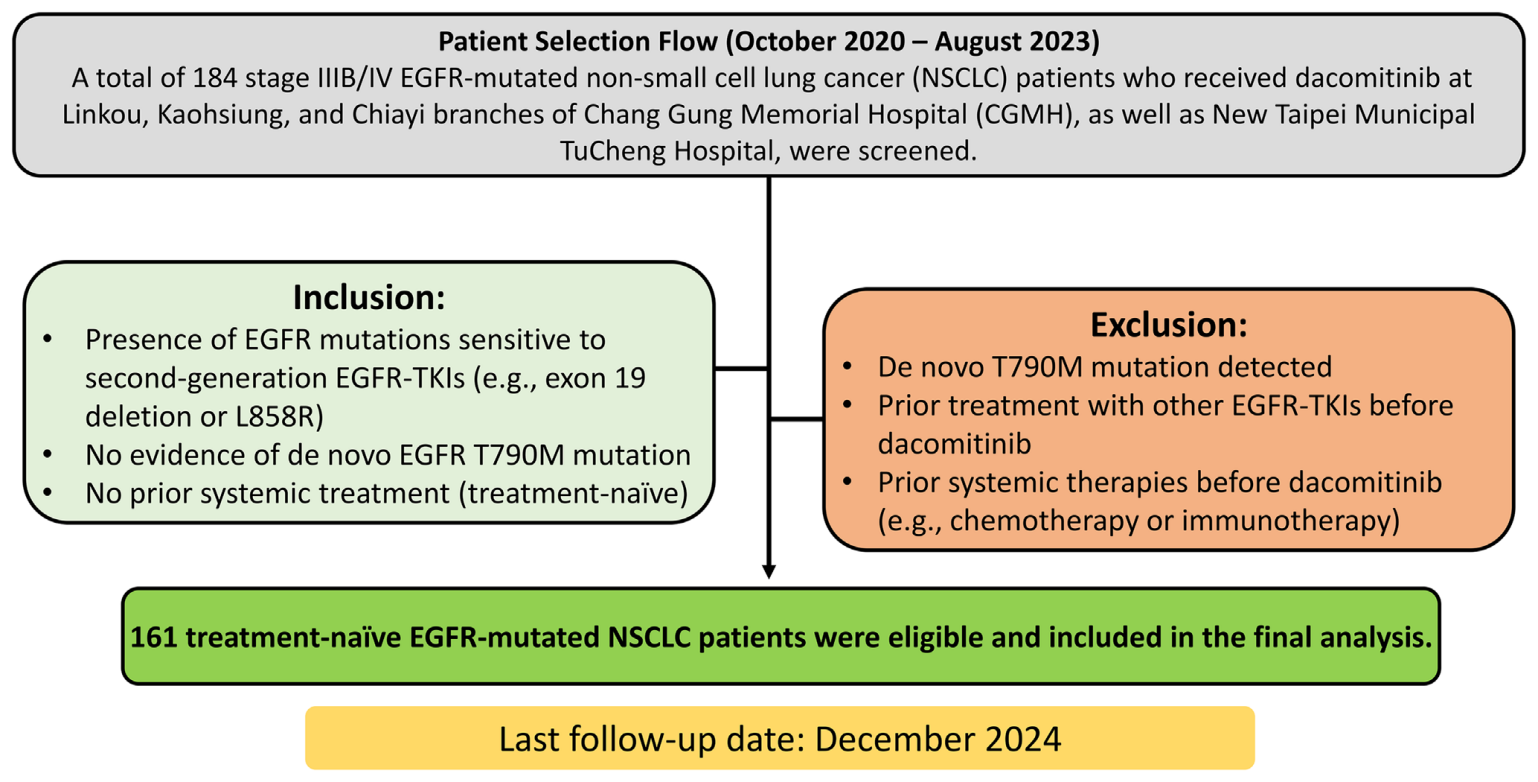
Scheme of patient selection process for the retrospective cohort with a predefined data cutoff on December 31, 2024.

Between October 2020 and August 2023, 184 histologically diagnosed stage IIIB/IV NSCLC patients with EGFR mutations who received dacomitinib were screened, and 161 patients were ultimately included for analysis. The patient selection process is summarized in Figure 1. The inclusion criteria were as follows: (1) EGFR mutation positive and sensitive to 1st‐ and 2nd‐generation EGFR‐TKIs such as exon 19 deletion and L858R; (2) no de novo EGFR T790M mutation; and (3) systemic treatment‐naïve (i.e., no targeted therapy including EGFR‐TKIs, chemotherapy, or immunotherapy prior to dacomitinib). The exclusion criteria were as follows: (1) de novo T790M mutation; (2) other EGFR‐TKIs, including gefitinib, erlotinib, afatinib, or osimertinib, prior to dacomitinib therapy; or (3) other systemic treatments, such as chemotherapy or immunotherapy, prior to treatment with dacomitinib.

Before initiation of dacomitinib, all patients underwent a comprehensive diagnostic workup for disease staging, including contrast‐enhanced computed tomography (CT), fluorodeoxyglucose (FDG) positron emission tomography (PET), and contrast‐enhanced brain magnetic resonance imaging (MRI). Follow‐up CT scans were performed every 3–4 months during dacomitinib treatment to assess the therapeutic response in all study patients. Additional imaging procedures, such as sonograms, plain chest films, FDG‐PET, or MRI, were ordered during follow‐up when deemed necessary by the physicians to aid in disease status evaluation.

Tumor response and disease progression during dacomitinib treatment were assessed by local investigators at each participating center according to the Response Evaluation Criteria in Solid Tumors (RECIST) version 3.0, based on routinely obtained imaging studies.

Responses were classified as complete (CR) and partial (PR); stable (SD) and progressive disease (PD) were considered to indicate no response to treatment. PFS was defined as the interval from the initiation of dacomitinib therapy to radiologically confirmed disease progression or death from any cause, whichever occurred first, with disease progression assessed by local investigators at each participating center. Overall survival (OS) was defined as the time from the start of dacomitinib treatment to death. Patients without documented progression or death by the last follow‐up date (December 31, 2024) were censored at their most recent clinical assessment. Treatment‐related adverse events (AEs) during first‐line dacomitinib therapy were reviewed from electronic patient charts and graded on the basis of the CTCAE v3.0 issued by the National Cancer Institute [20].

EGFR mutations, including primary or secondary mutations associated with resistance to first‐line dacomitinib therapy, were detected by using single‐nucleotide polymerase chain reaction (PCR), amplification refractory mutation system (ARMS)‐PCR, or next‐generation sequencing (NGS).

### 2.2 Statistical Analysis

The demographic and treatment‐related characteristics of the patients were summarized using quantitative measures. Age is expressed as medians and ranges. The impact of various clinical variables on PFS was assessed by using univariate and multivariate Cox proportional hazards models. The Kaplan–Meier method was used to estimate PFS and OS, and group comparisons were performed using the log‐rank test. A two‐tailed *p* value < 0.05 was considered to indicate statistical significance. All the statistical analyses were conducted using IBM SPSS Statistics software (version 22.0; SPSS Inc., Chicago, IL, USA). Survival plots for PFS and OS were generated using GraphPad Prism (version 5.0; GraphPad Software, San Diego, CA, USA).

## 3 Results

### 3.1 Patient Demographic Characteristics and Baseline Treatment Characteristics

Baseline patient demographics and treatment details are presented in Table 1. Regarding histology, 160 (99.4%) patients had adenocarcinoma, and 1 (0.6%) patient had NSCLC‐not otherwise specified (NSCLC‐NOS). Regarding the EGFR mutations, 49 (30.4%) patients had exon 19 mutations, and the other 112 (69.6%) patients had L858R mutations. Regarding the treatment dose adjustment, 60 (37.3%) patients had dose de‐escalation, and 4 (2.5%) had dose escalation. The reason for dose de‐escalation in 60 (37.3%) patients was concern over AEs. Dose titration (from 15 to 30 mg) was performed in 4 (2.5%) patients because of clinical considerations: 1 (0.6%) patient had an ECOG‐PS of 2, and the remaining 3 (1.9%) patients were over 80 years old. Thirty‐eight (23.6%) patients received early local therapies (within 30 days before or after initiating first‐line dacomitinib) in addition to first‐line dacomitinib therapy. Among these 38 (23.6%) patients, 34 (21.1%) received radiation therapy for bone metastasis, 2 (1.2%) received radiation therapy for intrathoracic tumors due to superior vena cava (SVC) syndrome, and 2 (1.2%) received thoracic surgery for diagnostic purposes. The use of additional local treatments did not differ significantly between the exon 19 deletion and L858R subgroups (Figure S2A; *p* = 0.528). Throughout the study period, none of the patients received dacomitinib in combination with anti‐angiogenic agents, such as bevacizumab or ramucirumab; all patients were treated with dacomitinib monotherapy in the first‐line setting. The median follow‐up time in this study was 22.20 months.

**TABLE 1:** Baseline demographic and clinical characteristics of the study cohort (*N* = 161).

Variable	N (%)
Sex	
Male	73 (45.3)
Female	88 (54.7)
Age, years (median/range)	67/36–88
ECOG performance status	
0–1	118 (73.3)
≥ 2	43 (26.7)
Smoking history	
Never‐smoker	123 (76.4)
Former/current smoker	38 (23.6)
Histology	
Adenocarcinoma	160 (99.4)
NSCLC, not otherwise specified	1 (0.6)
Clinical stage	
IIIB	5 (3.1)
IVa	85 (52.8)
IVb	71 (44.1)
EGFR mutation type	
Exon 19 deletion	49 (30.4)
L858R	112 (69.6)
Distant metastatic sites	
Bone	81 (50.3)
Adrenal	29 (18.0)
Liver	16 (9.9)
Initial dose reduction	60 (37.3)
45 → 30 mg	28 (17.4)
45 → 30 → 15 mg	8 (5.0)
30 → 15 mg	24 (14.9)
Dose re‐escalation	
15 → 30 mg	4 (2.5)
Early local therapy (±30 days from dacomitinib initiation)	38 (23.6)
Bone radiation	34 (21.1)
Thoracic radiation	2 (1.2)
Thoracic surgery	2 (1.2)
Median follow‐up duration, months	22.2

Abbreviations: ECOG PS, Eastern Cooperative Oncology Group performance status; EGFR, epidermal growth factor receptor; NSCLC, non‐small cell lung cancer.

### 3.2 Efficacy of Dacomitinib Therapy

**FIGURE 2:**
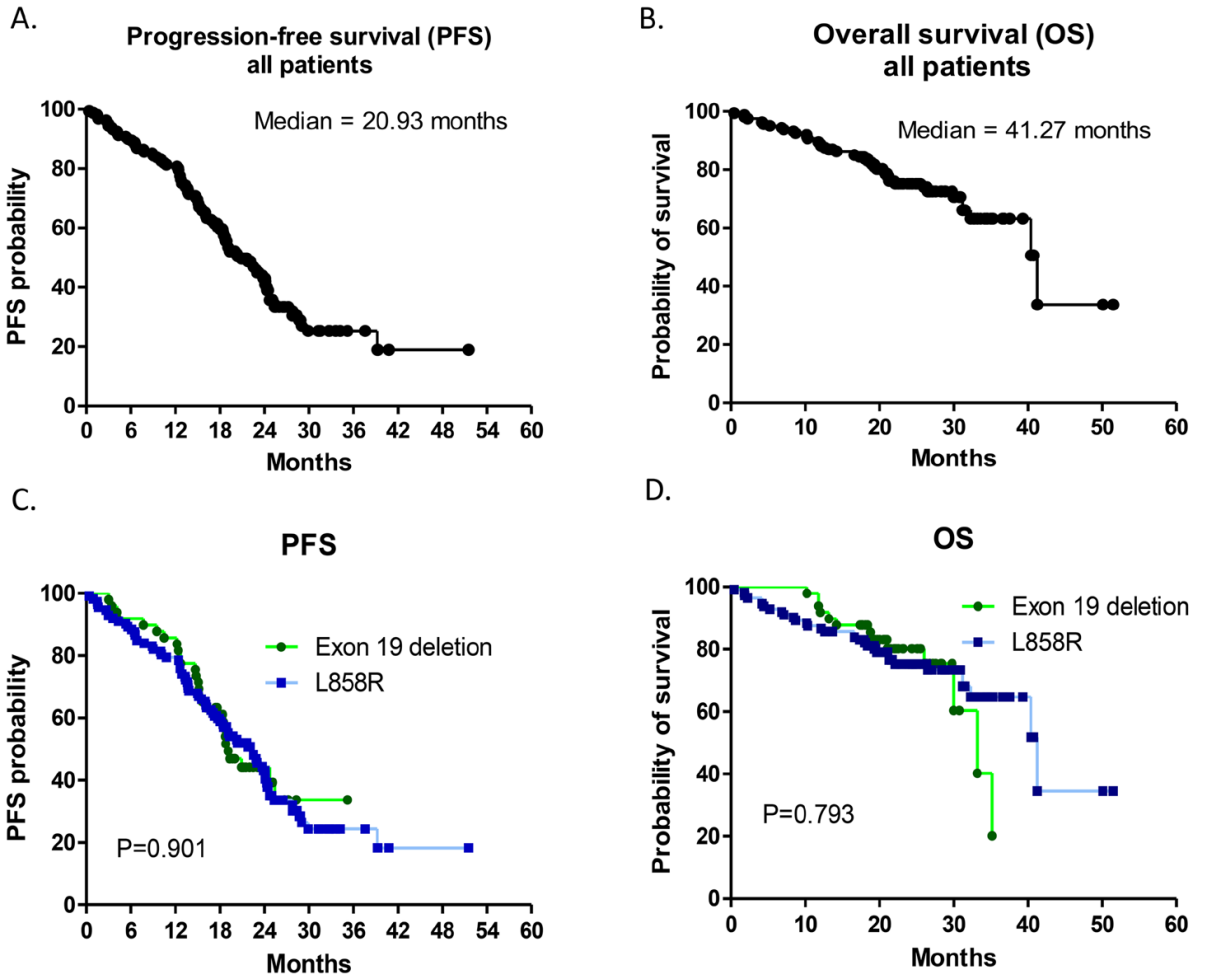
Kaplan–Meier survival curves showing the progression‐free survival (PFS) and overall survival (OS) outcomes of all study patients and subgroup analysis on the basis of EGFR mutation type. (A) Median PFS of all patients treated with first‐line dacomitinib. (B) Median OS of all patients treated with first‐line dacomitinib. (C) Comparison of the median PFS between patients harboring exon 19 deletions and those harboring L858R mutations. (D) Comparison of median OS between patients harboring exon 19 deletions and L858R mutations.

Among the 161 patients who received first‐line dacomitinib treatment, 1 (0.6%) achieved CR, 102 (63.4%) achieved PR, 44 (27.3%) had SD, and 14 (8.7%) had PD. The ORR reached 64.0%, while the DCR was 91.3% (as shown in Table 2). The median PFS was 20.93 months (95% confidence interval (CI), 17.55–24.32; Figure 2A), and the median OS was 41.27 months (95% CI, 31.71–50.82; Figure 2B) for all patients. The median PFS and OS were compared between patients with different EGFR mutations (exon 19 deletion and L858R) who received first‐line dacomitinib therapy. The median PFS was 22.53 months for patients with the L858R mutation and 19.10 months for those with the exon 19 deletion (hazard ratio [HR] = 0.973; 95% CI, 0.628–1.507; *p* = 0.901 via the log‐rank test; Figure 2C). The median OS was 41.27 months for patients with the L858R mutation and 33.17 months for those with exon 19 deletion (HR = 0.804; 95% CI, 0.279–1.329; *p* = 0.793 via the log‐rank test; Figure 2D). No statistically significant differences in either PFS or OS were observed between the two EGFR mutation groups.

**TABLE 2:** Efficacy outcomes of first‐line dacomitinib therapy (*N* = 161).

Efficacy parameter	*N* (%) or value
Complete response (CR)	1 (0.6)
Partial response (PR)	102 (63.4)
Stable disease (SD)	44 (27.3)
Progressive disease (PD)	14 (8.7)
Objective response rate (ORR)	64.0%
Disease control rate (DCR)	91.3%
Median progression‐free survival (PFS), months	20.93 (95% CI: 17.55–24.32)
Median overall survival (OS), months	41.27 (95% CI: 31.71–50.82)

Abbreviations: CI, confidence interval; CR, complete response; DCR, disease control rate; ORR, objective response rate; OS, overall survival; PD, progressive disease; PFS, progression‐free survival; PR, partial response; SD, stable disease.

### 3.3 Analysis of Predictive Factors for Progression‐Free Survival (PFS)

PFS was evaluated on the basis of various clinical variables using Cox regression analysis, as summarized in Table 3. In the univariate Cox regression analysis, baseline characteristics such as ECOG PS ≥ 2, stage IVb disease, and the presence of bone, adrenal, or liver metastases were significantly correlated with reduced PFS.

**TABLE 3:** Cox regression analysis for progression‐free survival in patients treated with dacomitinib.

Variables	Median PFS (months)	Univariate analysis		Multivariate analysis	
		HR (95% CI)	*p*	HR (95% CI)	*p*
Gender					
Male	24.10	reference	0.511		
Female	19.10	1.142 (0.769–1.694)			
Age					
< 70 years	19.10	reference	0.917		
≧ 70 years	22.90	0.978 (0.649–1.476)			
ECOG PS				1.776 (1.136–2.778)	0.012
0–1	23.90	reference	0.002		
≥ 2	17.07	2.000 (1.294–3.096)			
Smoking history					
Non‐smoker	22.53	reference	0.114		
Former/current smoker	19.10	1.422 (0.919–2.203)			
Stage					
IIIB	23.03	reference	0.013		
IVA	22.90				
IVB	15.50	1.645 (1.109–2.439)			
EGFR mutations					
Exon 19 deletion	19.10	reference	0.902		
L858R	22.53	1.028 (0.663–1.595)			
Metastatic sites					
Bone				1.515 (1.007–2.283)	0.046
Without bone metastasis	23.90	reference	0.006		
With bone metastasis	18.43	1.751 (1.178–2.604)			
Adrenal					
Without adrenal metastasis	23.03	reference	0.004		
With adrenal metastasis	12.23	2.045 (1.259–3.322)			
Liver				4.237 (2.358–7.576)	< 0.001
Without liver metastasis	23.70	reference	< 0.001		
With liver metastasis	9.03	5.348 (3.049–9.433)			
Dose reduction					
With dose reduction	23.03	reference	0.173		
Without dose reduction	19.10	1.329 (0.883–2.001)			
Early local therapy					
No	20.93	reference	0.371		
Yes	18.43	1.229 (0.782–1.933)			

Abbreviations: CI, confidence interval; ECOG PS, eastern cooperative oncology group performance status; EGFR, epidermal growth factor receptor; HR, hazard ratio; PFS, progression‐free survival.

**FIGURE 3:**
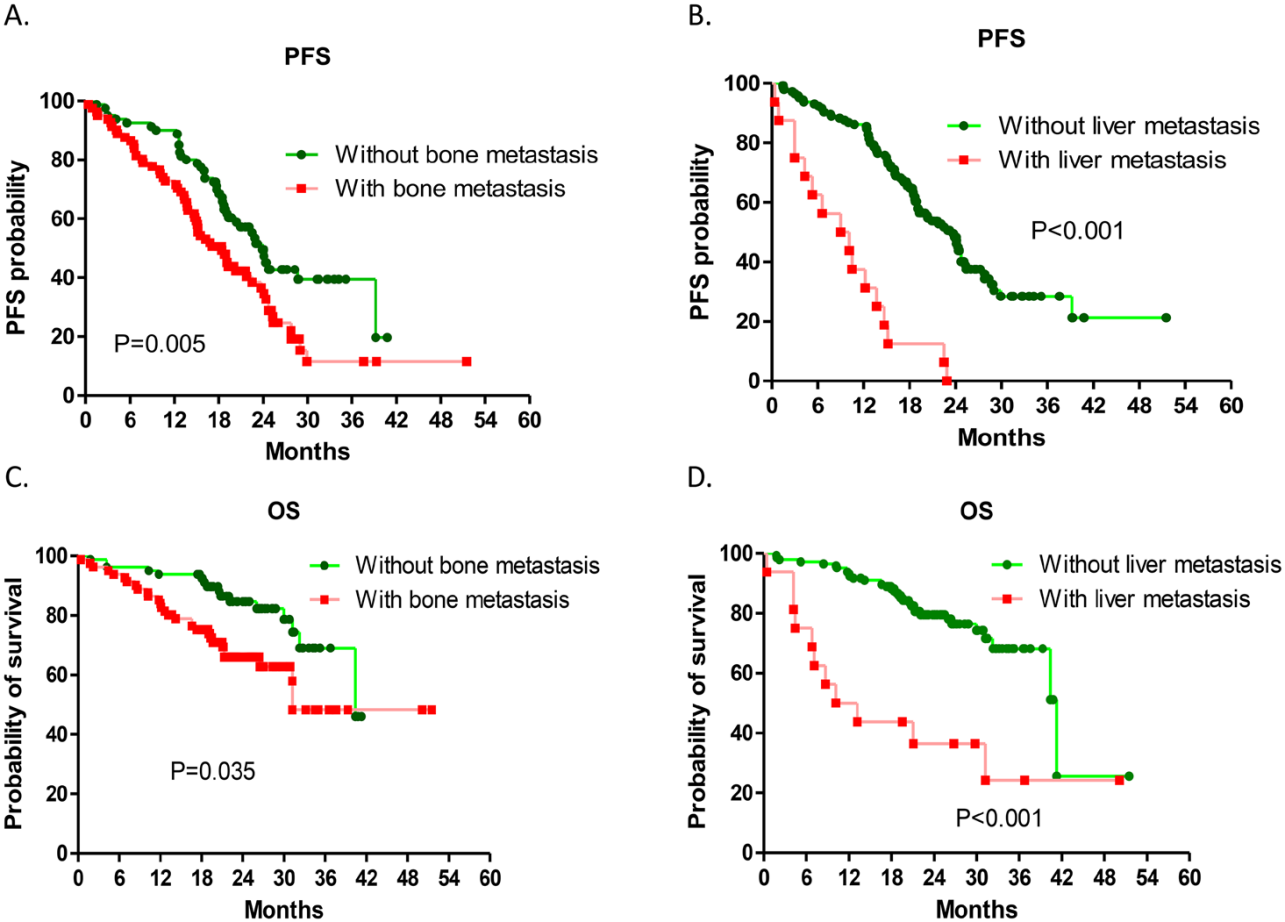
Kaplan–Meier survival curves showing the differences in progression‐free survival (PFS) and overall survival (OS) between patients with and without bone or liver metastases at baseline. (A) PFS comparison between patients with and without bone metastases (HR = 1.760; 95% CI: 1.346–2.611; *p* = 0.005, log‐rank test). (B) PFS comparison between patients with and without liver metastases (HR = 5.247; 95% CI: 3.002–9.170; *p* < 0.001, log‐rank test). (C) OS comparison between patients with and without bone metastases (HR = 2.185; 95% CI: 1.221–3.912; *p* = 0.004, log‐rank test). (D) OS comparison between patients with and without liver metastases (HR = 11.117; 95% CI: 3.663–33.738; *p* < 0.001, log‐rank test).

Multivariate Cox regression revealed that an ECOG PS ≥ 2 and the presence of bone and liver metastases were significant independent factors associated with decreased PFS. Patients were stratified on the basis of the presence or absence of bone and liver metastases, and PFS and OS were compared between these subgroups. Patients with bone metastasis had a significantly shorter PFS (18.43 vs. 23.90 months, HR = 1.760; 95% CI, 1.346–2.611; *p* = 0.005 via the log‐rank test; Figure 3A) and OS (31.23 vs. 40.40 months, HR = 2.185; 95% CI, 1.221–3.912; *p* = 0.004 via the log‐rank test; Figure 3C) than those without bone metastasis. Patients with liver metastasis had a significantly shorter PFS (9.58 vs. 23.70 months, HR = 5.247; 95% CI, 3.002–9.170; *p* < 0.001 via the log‐rank test; Figure 3B) and OS (11.68 vs. 41.27 months, HR = 11.117; 95% CI, 3.663–33.738; *p* < 0.001 via the log‐rank test; Figure 3D) than those without bone metastasis. Moreover, neither dose reduction nor the use of additional local treatments, such as radiotherapy, was associated with a significant effect on progression‐free survival (PFS) during first‐line dacomitinib therapy (Figures S1A and S2B). In addition, overall survival (OS) did not differ significantly between patients who did and did not undergo dose reduction (Figure S1B).

### 3.4 Clinical Characteristics and Subsequent Management After Disease Progression Following First‐Line Dacomitinib Therapy

A total of 101 (62.7%) patients experienced disease progression following first‐line dacomitinib therapy, and the details of the subsequent treatments are presented in Table 4.

**TABLE 4:** Post‐progression clinical information and therapies following first‐line dacomitinib treatment.

Parameter	*N* (%)
Non‐PD to dacomitinib	60 (37.3)
PD to dacomitinib	101 (62.7)
Sites of PD	
Lung	48 (47.5)
Mediastinal lymph nodes	4 (4.0)
Neck lymph nodes	2 (2.0)
Pleura	21 (20.8)
Pericardium	1 (1.0)
Peritoneum	1 (1.0)
Brain	3 (3.0)
Bone	12 (11.9)
Liver	9 (8.9)
EGFR‐T790M mutation testing after PD	85 (84.2)
Tissue rebiopsy	85 (84.2)
Circulating tumor (ct) DNA	3 (3.0)
EGFR‐T790M mutation result	
Positive	43 (50.6)
Negative	42 (49.4)
Positive rate	50.6%
Small cell transformation	1 (1.0)
Post‐progression systemic therapies	
Osimertinib	43 (42.6)
Dacomitinib	6 (5.9)
Chemotherapy	35 (34.7)
Platinum‐based doublet	22 (21.8)
Single agent chemotherapy	11 (10.9)
Anti‐angiogenesis agent (bevacizumab) + platinum‐based chemotherapy	2 (2.0)
Supportive care (no systemic treatment)	17 (16.8)

Abbreviations: ctDNA, circulating tumor DNA; EGFR, epidermal growth factor receptor; PD, progressive disease.

The lung was the most common site of disease progression (47.5%), followed by the pleura (20.8%), bone (11.9%), liver (8.9%), mediastinal lymph nodes (4.0%), and brain (3.0%). Additionally, 1 (2.0%) patients developed neck lymph node metastases, 1 (1.0%) patient had pericardial metastasis, and 1 (1.0%) patient had peritoneal metastasis at the time of progression. Among the 101 (62.7%) patients with disease progression, 85 (84.2%) underwent tissue rebiopsy for secondary T790M mutation tests, and 3 (3.0%) patients underwent both liquid biopsy (circulating tumor (ct)‐DNA) and tissue rebiopsies. Among the 85 patients who underwent rebiopsies for T790M mutation tests, the positive mutation rate was 50.6%. One (1%) patient was found to have small cell transformation on histology according to tissue rebiopsy. All patients with positive secondary T790M mutations received the 3rd‐generation EGFR‐TKI osimertinib as a second‐line therapy. Among the 6 (5.9%) patients who continued dacomitinib therapy, 4 (4.0%) received additional radiation therapy for newly developed bone metastases, whereas the remaining 2 (2.0%) underwent surgical resection for newly diagnosed pulmonary oligometastases.

Among the remaining 52 (51.5%) patients, 22 (21.8%) received platinum‐based doublet chemotherapy, and 11 (10.9%) received single‐agent chemotherapy following first‐line dacomitinib treatment. Two (2.0%) patients received a combination of bevacizumab, cisplatin, and pemetrexed as second‐line therapy. The remaining 17 (16.8%) patients did not receive further systemic anticancer therapy and were managed with supportive care.

### 3.5 Adverse Events (AEs) Associated With First‐Line Dacomitinib Treatment

The AEs associated with first‐line dacomitinib treatment are summarized in Table 5. Among the 161 patients in this study, the most frequent AE was skin toxicity (95.0%), followed by paronychia (92.5%), diarrhea (78.9%), stomatitis (63.4%), and nausea or vomiting (38.5%). Grade 3 AEs were included in the 5 AEs mentioned above. All grade 3 skin toxicity and paronychia were controlled by adjusting the dacomitinib dose and consulting with dermatologists for local treatments. Other grade 3 AEs in the gastrointestinal (GI) tract, including diarrhea, stomatitis, and nausea or vomiting, were controlled by reducing the dacomitinib dose or temporally interrupting dacomitinib therapy. Additionally, grade 3 diarrhea was controlled by increasing the loperamide dose. Nutrition and fluid supplements were administered to patients experiencing grade 3 stomatitis and nausea or vomiting. No AE‐related deaths were recorded in this study. The safety of dacomitinib as a first‐line therapy for advanced NSCLC patients with EGFR mutations was found to be acceptable, and treatment‐associated AEs were found to be manageable.

**TABLE 5:** Dacomitinib‐related adverse events.

Adverse event	All grades, *N* (%)	Grade 1–2, *N* (%)	Grade 3, *N* (%)	Grade 4, *N* (%)
Skin rash/acne	153 (95.0)	142 (88.2)	11 (6.8)	0
Paronychia	149 (92.5)	143 (88.8)	6 (3.7)	0
Diarrhea	127 (78.9)	114 (70.8)	13 (8.1)	0
Stomatitis	102 (63.4)	91 (56.6)	11 (6.8)	0
Nausea or vomiting	62 (38.5)	57 (35.4)	5 (3.1)	0
Constipation	4 (2.5)	4 (2.5)	0	0
Increased liver transaminases	10 (6.2)	10 (6.2)	0	0
Increased creatinine	17 (10.6)	17 (10.6)	0	0
Fever	2 (1.2)	2 (1.2)	0	0
Infection	2 (1.2)	2 (1.2)	0	0

## 4 Discussion

Our findings offer valuable perspectives on the real‐world clinical outcomes of first‐line dacomitinib treatment for advanced NSCLC with EGFR mutations. Our findings revealed that first‐line dacomitinib treatment yielded an ORR of 64.0% and a median PFS of 20.93 months. Multivariate Cox regression analysis revealed that an ECOG PS ≥ 2 and the presence of bone and liver metastases were independent factors associated with a shorter median PFS. The T790M mutation positivity rate was 50.6% among patients with acquired resistance to first‐line dacomitinib and patients who underwent rebiopsies for acquired T790M mutation tests. The safety profile of dacomitinib was deemed acceptable, with the majority of AEs being manageable in this study.

In two previous retrospective studies, dacomitinib was shown to have an ORR of approximately 80% and a median PFS of more than 16 months when used as first‐line therapy for advanced EGFR‐mutated NSCLC [21, 22]. Together with the results of our study, dacomitinib has been found to be an effective first‐line EGFR‐TKI for advanced EGFR‐mutated NSCLC in real‐world clinical practice. In the previous study by Shin et al., the sample size was 153, which is comparable to the sample size in our study [21]; furthermore, 45.5% of the patients in their study had baseline brain metastasis, while no patients had brain metastasis at baseline in the current study. Several previous studies reported that NSCLC patients with EGFR mutations and brain metastasis at baseline had shorter median PFS after treatment with EGFR‐TKI, and brain metastasis was identified as an unfavorable risk factor for clinical outcomes [23, 24, 25]. Additionally, 23.6% of the patients in our study received early local therapies such as radiation therapy or surgery during dacomitinib treatment. Altogether, these findings may explain why the PFS of patients receiving dacomitinib therapy in our study was longer than that reported in previous studies [22].

Osimertinib is a third‐generation EGFR‐TKI that forms an irreversible covalent bond with the tyrosine kinase domain of the EGFR, leading to sustained inhibition of EGFR signaling. In the FLAURA prospective clinical trial, osimertinib had a significantly longer median PFS than first‐generation EGFR‐TKIs (gefitinib and erlotinib) when used as first‐line therapies for EGFR‐mutated NSCLC (18.9 vs. 10.2 months). In the same clinical trial, osimertinib was shown to significantly improve OS compared with gefitinib or erlotinib [26]. According to the subgroup analysis of the FLAURA trial, osimertinib was strongly associated with OS in non‐Asian patients and those with exon 19 deletion [26]. Unlike the FLAURA trial, the OS benefit of first‐line dacomitinib was prominent in Asian patients and those with the L858R mutation in the ARCHER 1050 trial [16]. In our study, L858R‐mutated patients tended to have longer median OS than those with exon 19 deletion mutations (42.17 vs. 33.17 months). The results of our study are comparable with the results of the ARCHER 1050 trial [16].

Currently, osimertinib is the preferred EGFR‐TKI for the first‐line treatment of advanced EGFR‐mutated NSCLC based on the results of the FLAURA trial [11, 26]. However, in the analysis of previous real‐world clinical studies, first‐line osimertinib did not lead to improved OS compared with the use of 1st‐ or 2nd‐generation EGFR‐TKIs as first‐line therapy in EGFR‐mutated NSCLC patients [27, 28, 29, 30]. Some previous studies demonstrated that EGFR‐mutated NSCLC patients receiving first‐line treatment with 2nd‐generation EGFR‐TKI (afatinib) therapy followed by subsequent osimertinib had a median OS of approximately 50 months [17, 31, 32, 33]. Moreover, the cost‐effectiveness of first‐ to third‐generation EGFR‐TKIs remains a critical consideration in public healthcare systems. Despite its favorable efficacy and safety profile, the high cost of osimertinib may impose a substantial financial burden on national insurance programs in many countries. For patients without insurance coverage, the affordability of osimertinib also poses a significant barrier to access [34, 35]. In a previous phase 2B trial (LUX‐Lung 7), the 2nd‐generation EGFR‐TKI afatinib was shown to have a slightly longer PFS than gefitinib did (11.0 vs. 10.9 months) in first‐line therapy for EGFR‐mutated NSCLC [36]. In the LUX‐Lung 7 trial, first‐line afatinib ultimately did not yield a significant improvement in OS compared with the gefitinib group [37]. Therefore, dacomitinib continues to represent a reasonable first‐line therapeutic choice for patients with EGFR‐mutated NSCLC. First‐line dacomitinib was shown to have a survival benefit for certain subgroups of patients who are Asian or have the L858R mutation in the ACHER 1050 trial [16]. First‐line osimertinib was shown to have a survival benefit in non‐Asian patients and patients with exon 19 deletion mutations [27]. In the future, additional clinical studies may be warranted to determine whether first‐line dacomitinib or osimertinib is superior for treating EGFR‐mutated NSCLC patients and improving the clinical outcomes of these patient groups.

Previous studies have shown that patients with EGFR‐mutated NSCLC with baseline liver and bone metastases have shorter PFS and poor outcomes after treatment with first‐ and second‐generation EGFR‐TKIs [38, 39]. Our results showed that patients with baseline bone and liver metastasis had significantly shorter PFS after receiving first‐line dacomitinib therapy than patients without bone and liver metastasis. The results of our study revealed that baseline unfavorable factors were associated with clinical outcomes and were comparable with the results of previous studies [38, 39]. Additional strategies, such as combination therapy involving dacomitinib or other EGFR‐TKIs, need to be explored in patients with baseline unfavorable clinical factors.

The EGFR‐T790M point mutation, which is often referred to as a gatekeeper mutation within the tyrosine kinase domain of EGFR, is the most prevalent acquired resistance mechanism; it is observed in approximately 50% of patients harboring common activating EGFR mutations (such as exon 19 deletion and L858R) following treatment with first‐ and second‐generation EGFR‐TKIs [21, 22, 32, 33, 34]. The secondary T790M mutation positive rate was 50.6% in this study, which is consistent with the findings of previous studies [21, 22, 32, 33, 34]. All the study patients with acquired T790M mutation received osimertinib as second‐line therapy.

The dacomitinib treatment‐related AE profile recorded in our study is similar to the profile reported in previous clinical trials [10, 16]. Fortunately, the dacomitinib treatment‐related AEs reported in our study were manageable, and no treatment‐related deaths occurred. Grade 5 diarrhea was recorded in the ACHER 1050 trial. Dose de‐escalation was performed in 60 (37.3%) patientsin this study. Dose titration was performed in 4 (2.5%) study patients when these patients tolerated low‐dose dacomitinib well. According to the results of the ACHER 1050, adjusting the dose of dacomitinib is acceptable and does not negatively affect the efficacy of dacomitinib in advanced EGFR‐mutated NSCLC patients [10, 16].

This study has several limitations that need to be addressed. First, no patients with baseline brain metastasis were included in this study. Second, the primary EGFR mutations in all the study patients were either exon 19 deletion or L858R, and none of the patients with uncommon mutations, such as G719X, S768I, or L861Q, were included in this study. Third, although EGFR mutation subtype–based analyses were performed, comprehensive co‐mutation profiling (e.g., TP53 status) was unavailable for most patients due to the use of single‐gene EGFR PCR testing in routine practice. Residual confounding related to unmeasured co‐mutations cannot be entirely excluded. In accordance with the eligibility criteria of the ACHER 1050 trial, subjects with common EGFR mutations (exon 19 deletion and L858R) and without baseline central nervous system (CNS) metastasis were eligible for inclusion [10, 14]. First‐line dacomitinib therapy is recommended for non‐CNS metastatic NSCLC patients harboring common EGFR mutations. Additionally, the Taiwan Health Insurance Bureau's reimbursement policies for 1st‐ to 3rd‐generation EGFR‐TKIs usually depends on the results of previous prospective clinical trials. For example, the cost of afatinib is covered by the Taiwan Health Insurance Bureau, and this drug preferred for advanced NSCLC patients with major uncommon EGFR mutations (G719X/L861Q/S768I) [38]. For EGFR‐mutated NSCLC patients with baseline brain metastasis, afatinib and osimertinib are recommended as first‐line EGFR‐TKIs in Taiwan on the basis of evidence from previous clinical trial reimbursement policies [19, 40]. Together, these findings explain why non‐CNS metastatic NSCLC patients harboring common EGFR mutations were included in the current study. In our study, only 3 (3%) patients experienced disease progression involving brain metastases during first‐line dacomitinib treatment. Brain MRI is a highly sensitive modality for identifying brain metastases and is capable of detecting small, asymptomatic intracranial lesions in patients with lung cancer [41, 42]. However, routine brain MRI surveillance was not performed during first‐line dacomitinib treatment in this study. As a result, early asymptomatic intracranial progression may have gone undetected. Consequently, the ORR and PFS might be overestimated compared with those reported in an unselected real‐world population. The efficacy of first‐line dacomitinib in advanced NSCLC patients harboring uncommon EGFR mutations or baseline brain metastasis warrants exploration in future clinical studies.

## 5 Conclusion

Dacomitinib has favorable efficacy and tolerability as a first‐line treatment for advanced NSCLC without brain metastases and with common EGFR mutations, including exon 19 deletion and L858R. The presence of baseline bone and liver metastases was linked to poorer clinical outcomes, suggesting that additional combination strategies may be warranted for these subgroups.

Although prior studies have consistently shown better treatment outcomes in patients with EGFR exon 19 deletion than in those with L858R mutation when treated with EGFR‐TKIs, our cohort demonstrated a numerically longer OS in patients with the L858R mutation. This finding may be attributed to differences in baseline characteristics, post‐progression treatments, or small sample sizes. Further studies are warranted to validate whether dacomitinib offers differential benefits on the basis of the EGFR mutation subtype.

## Author Contributions

This manuscript has been read and approved by all authors, each of whom meets the ICMJE criteria for authorship. Conception and design of the study: P.‐C.H., H.‐W.K., and C.‐T.Y. Writing of the manuscript: P.‐C.H., H.‐W.K., L.‐C.C., and C.‐T.Y. Acquisition of the study data (information of the study patients): P.‐C.H., H.‐W.K., L.‐C.C., S.‐H.H., C.‐S.L., Y.‐C.L., S.C.‐H.K., J.‐S.J., C.‐C.W., and C.‐T.Y. Analysis and interpretation of the data: P.‐C.H., L.‐C.C., S.‐H.H., C.‐S.L., Y.‐C.L., S.C.‐H.K., and J.‐S.J. Validation of the data: P.‐C.H., H.‐W.K., and C.‐T.Y. Supervision of study work (review and revision of the study): P.‐C.H., C.‐T.Y., and C.‐C.W. Administrative and funding support: P.‐C.H.

## Funding

This study was supported by the Chang Gung Medical Research Project (grant no. CMRPG3N1331).

## Ethics Statement

This study was approved by the Institutional Review Board of the Chang Gung Medical Foundation (approval number: 202401686B0). Owing to its retrospective design, the requirement for informed consent was waived by the IRB. All study procedures were conducted in compliance with the principles outlined in the Declaration of Helsinki. No identifiable personal information, including patient IDs or birth dates, was disclosed in this manuscript.

## Conflicts of Interest

The authors declare no conflicts of interest.

## Supporting information


**Figure S1:** Kaplan–Meier analyses of progression‐free survival (PFS) and overall survival (OS) according to dose reduction status during first‐line dacomitinib treatment. (A) PFS comparison between patients with and without dose reduction (HR = 1.321; 95% CI, 0.887–1.969; *p* = 0.170). (B) OS comparison between patients with and without dose reduction (HR = 1.229; 95% CI, 0.673–2.244; *p* = 0.503).

**Figure S2:** (A) Distribution of additional local therapies according to EGFR mutation subtype (exon 19 deletion vs. L858R). No significant difference was observed between the two groups (*p* = 0.528). (B) Kaplan–Meier analysis of progression‐free survival (PFS) comparing patients who did and did not receive additional local therapy during first‐line dacomitinib treatment (HR = 0.803; 95% CI, 0.497–1.296; *p* = 0.369).

## Data Availability

The datasets generated and analyzed during the current study are not publicly available due to local regulations and institutional policies concerning patient confidentiality and data protection.
