# Supplementary figures and images for: Dacomitinib as a First‐Line Therapy for Advanced EGFR‐Mutated Non‐Small Cell Lung Cancer Without Brain Metastases: A Multicenter Retrospective Observational Study
Source: Cancer Med. 2026 Feb 27;15(3):e71659. doi: 10.1002/cam4.71659 (PMC12948714; doi:10.1002/cam4.71659)

A.

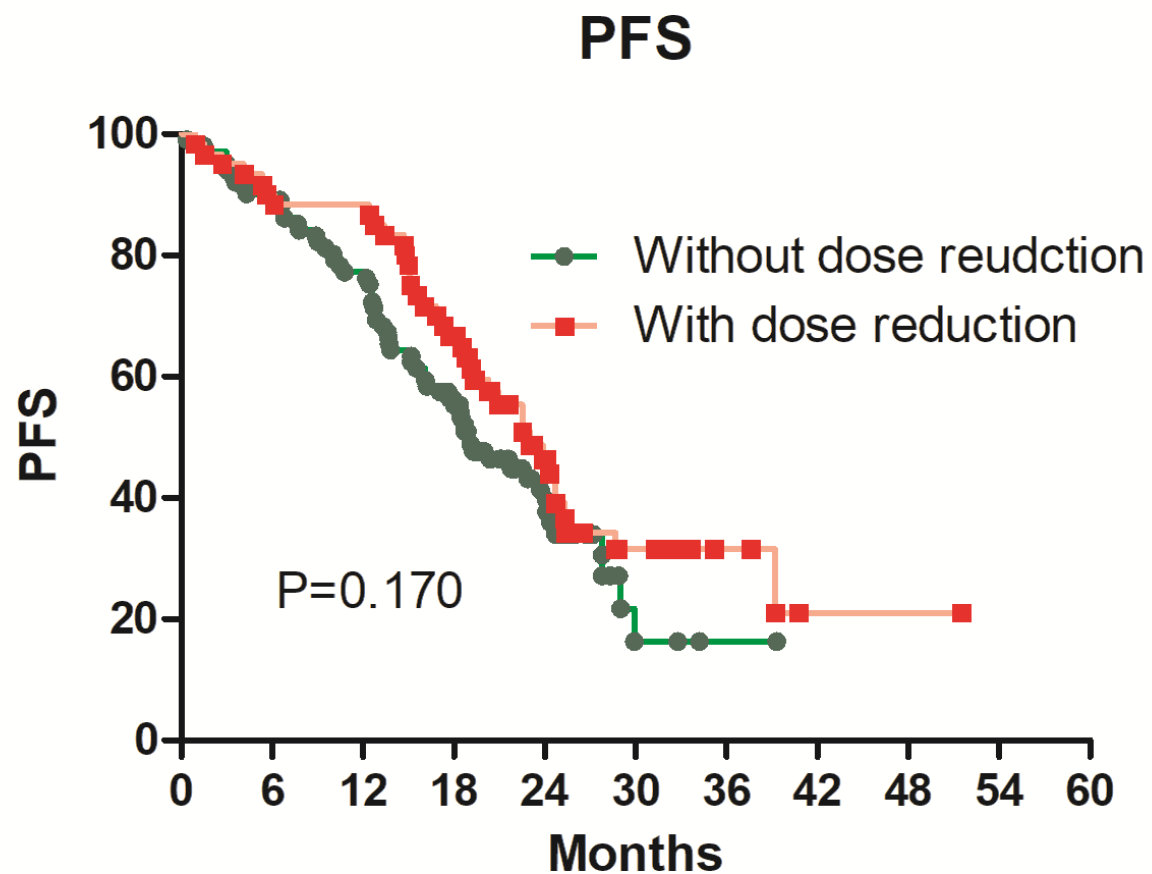

B.

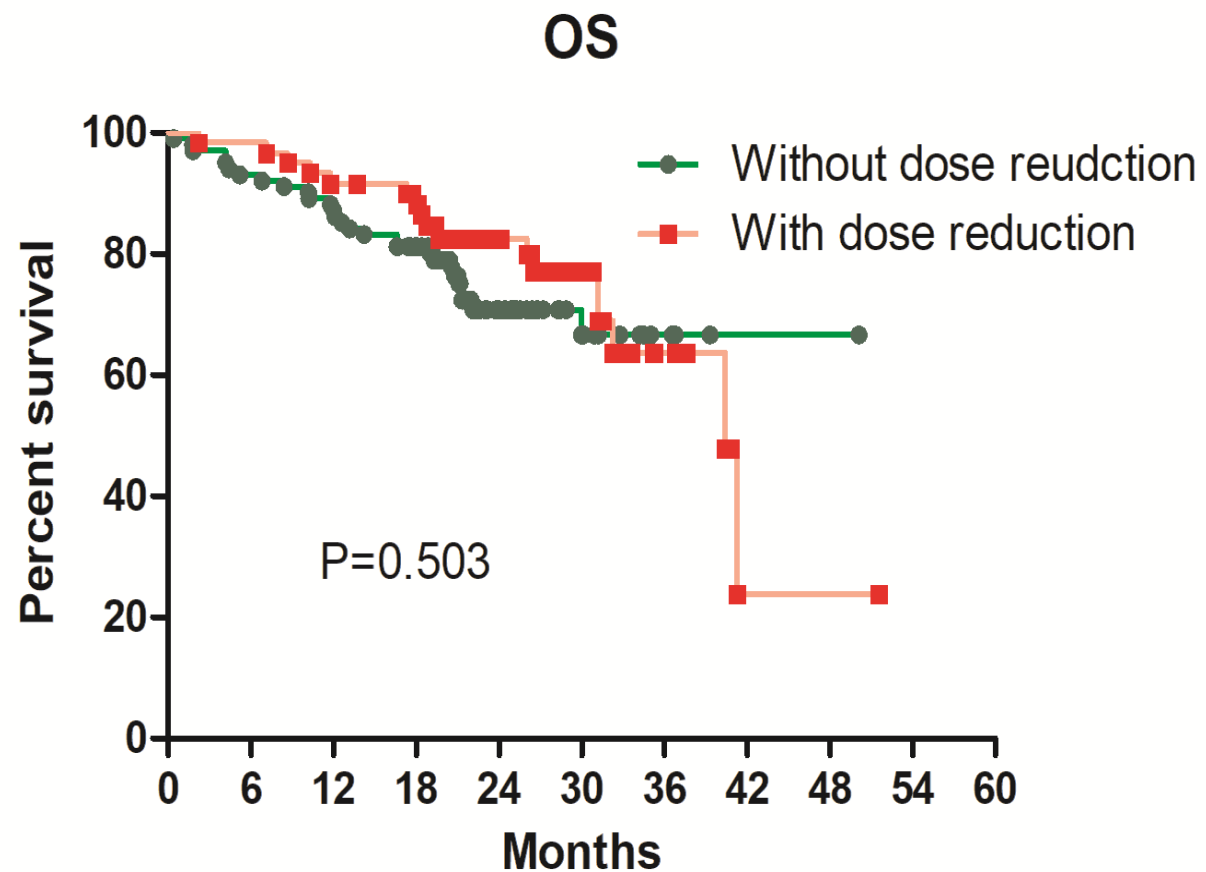

Supplement: Supplementary file 1 — Figure S1: Kaplan–Meier analyses of progression‐free survival (PFS) and overall survival (OS) according to dose reduction status during first‐line dacomitinib treatment. (A) PFS comparison between patients with and without dose reduction (HR = 1.321; 95% CI, 0.887–1.969; p = 0.170). (B) OS comparison between patients with and without dose reduction (HR = 1.229; 95% CI, 0.673–2.244; p = 0.503). [file CAM4-15-e71659-s002.pdf]

A.

|                  | With local therapy<br>N=38 | Without local therapy<br>N=123 | <i>p</i> -Value |
|------------------|----------------------------|--------------------------------|-----------------|
| Exon 19 deletion | 10 (26.3%)                 | 39 (31.7%)                     | 0.528           |
| L858R            | 28 (73.7%)                 | 84 (68.3%)                     |                 |

B.

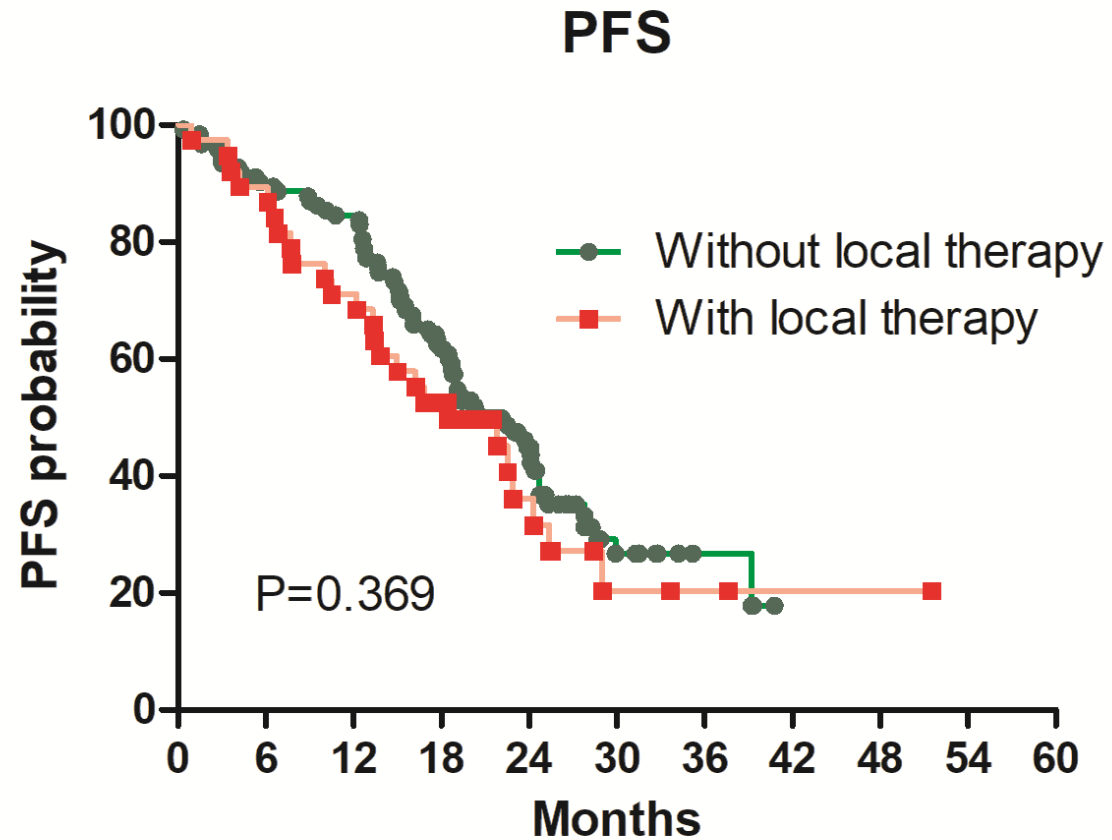

Supplement: Supplementary file 2 — Figure S2: (A) Distribution of additional local therapies according to EGFR mutation subtype (exon 19 deletion vs. L858R). No significant difference was observed between the two groups (p = 0.528). (B) Kaplan–Meier analysis of progression‐free survival (PFS) comparing patients who did and did not receive additional local therapy during first‐line dacomitinib treatment (HR = 0.803; 95% CI, 0.497–1.296; p = 0.369). [file CAM4-15-e71659-s001.pdf]
